# The corneal biomechanical changes of phacoemulsification in cataract patients: A systematic review and meta-analysis

**DOI:** 10.1371/journal.pone.0317179

**Published:** 2025-01-24

**Authors:** Xinru Li, Xiaofeng Li, Lintong Tan, Hu Chen, Yuan Lai

**Affiliations:** 1 Department of Ophthalmology, The First People’s Hospital of Yongkang Affiliated to Hangzhou Medical College, Yongkang, Zhejiang, P. R. China; 2 School of Ophthalmology and Optometry, Wenzhou Medical University, Zhejiang, P. R. China; University of Warmia, POLAND

## Abstract

**Purpose:**

To evaluate the corneal biomechanical properties of phacoemulsification in the treatment of cataract patients.

**Methods:**

Pertinent studies were searched in PubMed, EMBASE, Web of Science and clinicaltrials.gov., as of November 05, 2024. The reference lists of related published reviews were included as well. This meta-analysis was performed with Stata Software and Review Manager, we used mean difference (MD) to evaluate the statistical consequence, using I^2^ statistic to assess the heterogeneity. Subgroup analysis were performed under the occurrences of high heterogeneity. We used eleven items to describe the characteristics of included studies, publication bias was performed with Egger’s test. The quality assessment were evaluated with 3 items by Newcastle-Ottawa Scale (NOS) items.

**Results:**

Thirteen eligible studies were identified for data synthesis and assessment. According to the result of meta-analysis, the central corneal thickness(CCT) (MD = 10.50, 95% CI: [5.01, 15.98]; P<0.05) and intraocular pressure(IOPg)(MD = -0.73, 95% CI: [-1.26, -0.19]; P<0.05) of cataract patients after phacoemulsification was significantly higher than the control groups. The values of corneal hysteresis(CH) (MD = -0.43, 95% CI: [-0.62, -0.23]; P<0.05) and corneal resistance factor(CRF) (MD = -0.49, 95% CI: [-0.64, -0.33]; P<0.05) after phacoemulsification surgery were statistically lower than the control groups. While the values of IOPcc did not show statistically different (MD = -0.13, 95% CI: [-0.67, 0.41]; P = 0.64).

**Conclusion:**

Included data analysis indicated that the values of CCT, CH, CRF and IOPg showed statistical change in cataract patients after phacoemulsification surgeries compared with control groups. There is a correlation between corneal biomechanics and phacoemulsification surgeries.

## Introduction

Cataract is an ocular disease characterized by the opacification of the lens, with the loss of vision. The most common and effective treatment is the surgical removal of cataracts [1]. Phacoemulsification surgery is one of the most common eye surgeries. And it is one of the most frequently used ocular surgeries proven to be a safe and effective way to restore the clear vision, to enhance the quality of life. Phacoemulsification is the procedure of choice for the surgical management of cataract, with using the intraocular lens to replace the opaque lens. By the continuous development of surgical skills, the corneal incision is becoming smaller, and femtosecond lasers were used in cataract surgery [2].

Corneal biomechanics played a crucial role in maintaining viscoelasticity and shape [3]. The recent studies have demonstrated that corneal biomechanics has clinical significance for glaucoma, keratoconus, blood glucose detection and thyroid eye diseases [4–7]. Diseases, corneal surgeries or interventions might affect the corneal biomechanics [8]. And the impact of cataract surgeries could not be underestimated, either. By the way, phacoemulsification occurs in the confined space which close to the cornea, and the corneal biomechanical properties might change.

Various factors can induce corneal changes in cataract surgeries, such as the incision width and location. It has been reported that decreased incision width could reduce the corneal higher- order aberrations and corneal astigmatism [9,10]. While sometimes corneal changes induced by surgery were more substantial than expected. The corneal changes might influence the vision and satisfaction of patients [11].

The smaller incision allows for the decreased risk of prolapse of the iris, and maintenance of anterior chamber during surgeries. The smaller incision also means the fewer complications, like the low intraocular pressure, shallow anterior chamber and choroidal detachment. Viscoelastics, retained cataract fragments, surgical errors and the high phacoemulsification ultrasound energy all can cause the corneal oedema, particularly if the corneal endothelium existed abnormalities.

The intraocular pressure (IOP), central corneal thickness (CCT) or other index could be influenced, then cause the biomechanics data changed. Kamiya et al. reported that CCT influenced the corneal biomechanical properties, both corneal resistance factor(CRF) and corneal hysteresis(CH) recovered control group values in seven days after cataract surgery [12].

The study aimed to evaluate the corneal biomechanical changes in the phacoemulsification patients with cataract. According to the analysis of results, it can provide more instructives for clinical diagnosis and treatment.

## Methods

### Data source and search strategy

A literature search was conducted in PubMed, EMBASE, Web of Science and clinicaltrials. gov. Search syntax was (corneal biomechanics OR corneal hysteresis OR corneal biomechanical ocular response analyzer OR corneal resistance factor) AND (phacoemulsification OR cataract surgery OR femtosecond laser-assisted cataract surgery (FLACS)). References of included studies and related reviews were also searched. The included publications were limited in English, without publication time limit. Missing data were excluded from the analyses. The search was performed on November 05, 2024. Two reviewers searched and collected the articles, the other two reviewers screened and eliminated the duplicate publications independently. The disputes were resolved by discussing with the supervisor.

### Study seletion

#### Inclusion criteria

1. Qualification and diagnosis for cataract surgery;
2. Patients aged over 18 years old;
3. Patients had phacoemulsification cataract extraction surgeries or combined with FLACS;
4. No intraoperative complications or intravitreal treatment.

#### Exclusion criteria

1. Glaucoma, previous glaucoma surgery or using the pressure-lowering drops;
2. Corneal dystrophies or degenerations, corneal scarring, previous corneal surgery;
3. Congenital or traumatic cataracts;
4. Other eye surgeries or trauma.

### Data collection and quality assessment

Two reviewers screened titles and abstracts independently to obtain the eligible articles. The other two reviewers eliminated the duplicate publications. When the articles used the same measured items and data, we only included the latest report. If the subjects or indicators were different in various publications with using the same set of data, we still included to analyse.

Reviewers extracted relevant study characteristics from included articles (author, study design, country, ethnicity, gender, age, the number of included eyes, surgical technique, the size and direction of corneal incision, postoperative medication, the mode of operation and the machine type of measurement). Controversy between individual views was resolved through the discussion with the supervisor. Newcastle-Ottawa Scale (NOS) was used to assess the quality of included studies, more stars indicated higher scores, and it means the corresponding study had higher quality.

### Statistical analysi*s*

The statistical analysis was performed using Stata Software (version 15.0; Stata Corp LP, College Station, TX) and Review Manager (version 5.4; Cochrane Collaboration). We analyzed the correlation between phacoemulsification and corneal biomechanics in cataract patients, calculated the mean difference (MD) and 95% CIs for various groups in CCT, CH, CRF, IOPcc and IOPg. All the values were analyzed by using the random-effects model. The heterogeneity was statistically assessed by I^2^ statistics. And the I^2^ statistic <25% indicated low heterogeneity, 25%-50% indicated moderate heterogeneity, >50% indicated high heterogeneity. If high heterogeneity was observed, we would perform subgroup analysis to analyze. The characteristics of included studies were assessed by 12 items. Sensitivity analysis was used to evaluate whether the result were affected by the single study. Publication bias was performed with Egger’s test. If P <0.05, the difference between groups was statistically significant.

## Results

### Literature search

**Fig 1:**
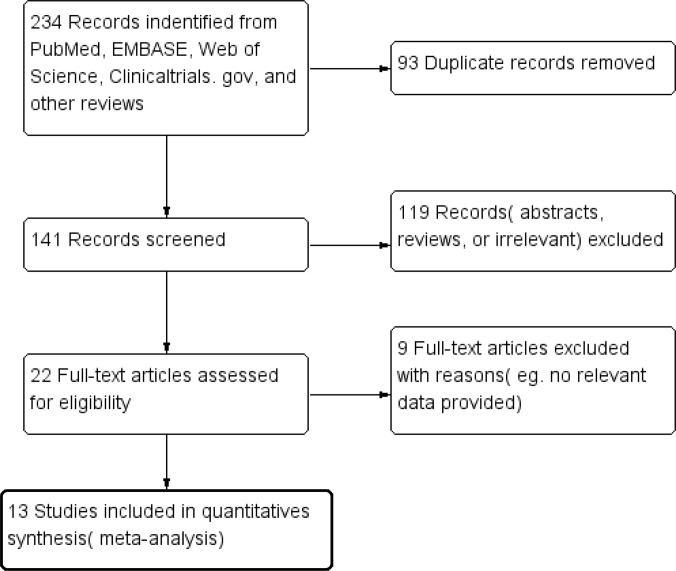
PRISM Flow Diagram of the literature search process.

We identified 234 papers through literature searches. And ninety-three relevant studies were included after the initial screening of duplicate studies. One hundred and nineteen studies were removed which not fulfilled the inclusion criteria, and 141 studies were included. After excluding the studies which could not provide the relevant or valid data, 13 observational studies constituted the data for analysis [11,13–25] (Fig 1).

### Study characteristics

The study characteristics were shown in Table 1. There were totally 13 included articles, eleven publications were prospective studies, two publications were retrospective studies. The 13 studies were chosen from 13 laboratories in eleven countries. Only one study stated the ethnicity. Ten studies had both male and female cataract patients, three publications did not stated the sex of patients. Two studies did not mention the age of patients. All of the included studies used the phacoemulsification and IOL implanation, and one study combined with the surgical skill of FLACS, two studies used the surgical skills of micro-incision phacoemulsification, two studies compared the corneal index of micro-incision and common incision. Four articles stated the direction of main incision, two studies ruled the definite position of corneal incision, one study chosen the direction of incision through corneal axis. Six studies showed the postoperative medication. There were two publications used the Corvis ST machines to measure the related corneal index, one publication used the pentacam machine, and 10 publications used ORA machines.

**Table 1:** Characteristics of included studies in the meta-analysis.

Author, Year	Study	Country	Ethnicity	Gender	Age	Sample size(eye)	Surgical technique	The size of corneal incision/mm	The direction of main corneal incision	Postoperative medication	The mode of operation	Machine	The time of measurement
*Pniakowska, 2019 [13]	Prospective clinical study	Poland	-	M/F	Group 1:(72.04±8.97)/Group 2:(72.41±10.47)	Group 1: 51/ Group 2:46	Coaxial micro-incision phacoemulsification+ IOL implanation	2.0	12 o’clock	-	PHACO	ORA	1 week/1 month
Hirasawa, 2018 [14]	Prospective clinical study	Japan	-	M/F	(73.3±6.6)	39	Phacoemulsification+ IOL implanation	2.8	-	Steroidal(0.1% Flumetholon), antibiotic(1.5% Cravit), nonsteroidal anti-inflammatory(0.1% Nevanac)	PHACO	Corvis ST	1 week/1 month/3 months
*Beato, 2020 [16]	Prospective clinical study	Portugal	-	M/F	Group 1:(72.8±5.8)/Group 2:(70.6±6.3)	Group 1: 44/ Group 2:44	Phacoemulsification+ IOL implanation	2.75	-	Dexamethasone, flurbiprofen, levofloxacin eye drops	PHACO	ORA	1 month/6 months
Kucumen, 2008 [19]	Prospective clinical study	Turkey	-	-	-	51	Microcoaxial phacoemulsification+ IOL implanation	2.4	-	-	PHACO	ORA	1 week/1 month/3 months
Kandarakis, 2012 [20]	Prospective clinical study	Greece	-	M/F	(73.9±7.4)	41	Phacoemulsification+ IOL implanation	2.75	-	-	PHACO	ORA	1 day/1 week
Hamoudi, 2017 [21]	Prospective clinical study	Denmark	-	-	-	20	Phacoemulsification+ IOL implanation	2.4	-	Nepafenac, a combination of dexamethasone/chloramphenicol	PHACO	Pentacam	1 month/3 months/12 months
*Alió, 2010 [23]	Prospective clinical study	Spain	-	M/F	Group 1:(72.37±10.27)/Group 2:(65.65±10.40)	Group1:30/ Group 2:30	Group 1: microincision cataract surgery/ Group 2: coaxial phacoemulsification	Group 1: 1.80/ Group 2: 2.75	The positive meridian of the astigmatism	0.3% ofloxacin, 0.1% dexamethasone alcohol	PHACO	ORA	Immediately/1 month
Kamiya, 2011 [22]	Retrospective clinical study	Japan	-	M/F	(69.4±9.40)	38	Phacoemulsification+ IOL implanation	2.8	The steepest corneal axis at the limbus	Steroidal(0.1% betamethasone), antibiotic(levofloxacin), diclofenac sodium(0.1% Diclod)	PHACO	ORA	1 day/1 week/1 month/3 months
Deol, 2015 [24]	Retrospective clinical study	American	Hispanic/Asian/White	M/F	(70.8±8.63)	65	Phacoemulsification+ IOL implanation	-	-	-	PHACO	ORA	2–4 months/10-12 months
Denoyer, 2013 [11]	Prospective clinical study	France	-	-	(71.0±8.0)	40	Phacoemulsification+ IOL implanation	2.75	-	-	PHACO	ORA	1 day
*Zhang, 2016 [17]	Prospective clinical study	China	-	M/F	Group 1: (69.7±11.3)/ Group 2: (70.1±10.2)	Group 1: 33/ Group 2: 35	Group 1: coaxial microincision phacoemulsification/ Group 2: standard incision phacoemulsification	Group 1: 2.2/Group 2: 3.0	10 o’clock	-	PHACO	ORA	1 day/1 week/2 weeks/3 weeks/4 weeks
Pakravan, 2014 [18]	Prospective clinical study	Iran	-	M/F	(66.2±16.2)	26	Phacoemulsification+ IOL implanation	2.8	-	0.5% topical chloramphenicol, 0.1% topical betamethasone	PHACO	ORA	3 months
*Wei, 2017 [25]	Prospective clinical study	China	-	M/F	Group 1: (63.79±7.38)/ Group 2: (67.21±9.34)	Group 1: 12/ Group 2: 38	Group 1: femtosecond laser-assisted cataract surgery and phacoemulsification/ Group 2: standard incision phacoemulsification	3.0	-	-	FLACS	Corvis ST	1 week/1 month

*Pniakowska, 2019 [13]: There were two groups were included in the study, the Group 1 included patients with corneal astigmatism of K1-K2< +1.0D; the Group 2 included patients with corneal astigmatism of K1-K2≤ +2.25D.

-: Not mentioned.

*Beato, 2020 [16]: There were two groups were included in the study, the Group 1 included patients with diabetic; the Group 2 included patients without diabetic.

*Alió, 2010 [23]: There were two groups were included in the study, the Group 1 included patients with microincision biaxial bimanual phacoemulsification; the Group 2 included patients with standard coaxial phacoemulsification.

*Zhang, 2016 [17]: There were two groups were included in the study, the Group 1 included patients with coaxial microincision phacoemulsification; the Group 2 included patients with standard incision phacoemulsification.

*Wei, 2017 [25]: There were two groups were included in the study, the Group 1 included patients with femtosecond laser-assisted cataract surgery and phacoemulsification; the Group 2 included patients with standard incision phacoemulsification.

PHACO: The surgical method of patients was phacoemulsification.

FLACS: The surgical method of patients was femtosecond laser-assisted cataract surgery and phacoemulsification.

ORA: The Ocular Response Analyzer.

## Comparison analysis

### CCT of cataract patients with phacoemulsification

**Fig 2:**
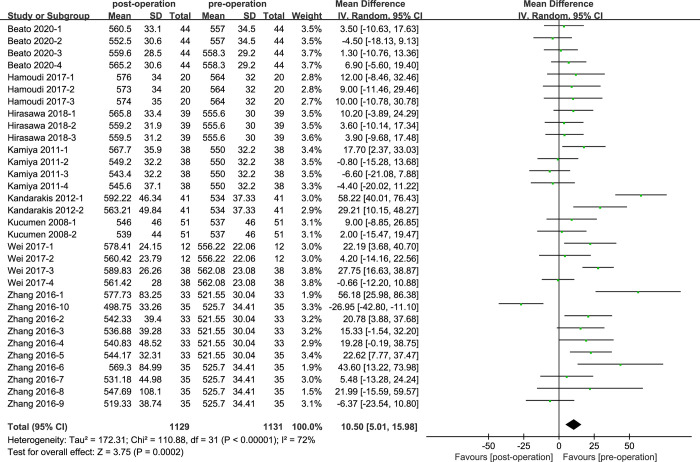
Comparison of CCT values.

A comparison was conducted on the CCT values between postoperative cataract patients and control group, as by eight of the 13 studies (Fig 2). According to the analysis, the heterogeneity was statistically high (I^2^ = 72%; P<0.05). The results showed that the CCT values of cataract phacoemulsification surgeries were statistically significant (MD = 10.50, 95% CI: [5.01, 15.98]; P<0.05).

### CH of cataract patients with phacoemulsification

**Fig 3:**
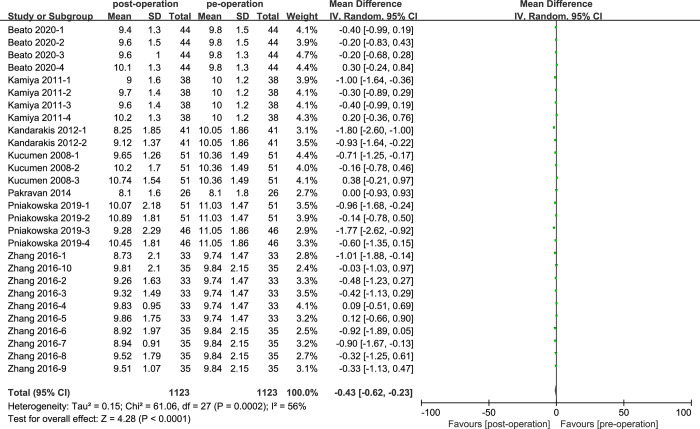
Comparison of CH values.

Seven articles were included to compare the CH of cataract patients before and after surgeries (Fig 3), there were totally 28 groups included to analyze. The CH values of cataract patients showed statistically significant (MD = -0.43, 95% CI: [-0.62, -0.23]; P<0.05). And the data of CH values comparisons were moderate heterogeneous (I^2^ = 56%; P<0.05).

### CRF of cataract patients with phacoemulsification

**Fig 4:**
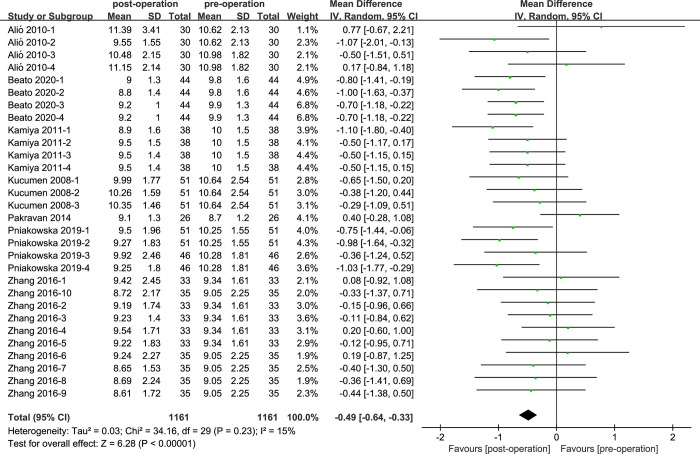
Comparison of CRF values.

There were 7 studies included in the comparison of CRF values (Fig 4). The heterogeneity was low (I^2^ = 15%; P = 0.23). Comparing the CRF values between pre-operation and post-operation of cataract surgeries, the result was showed that the CRF of cataract surgery patients was statistically lower than cataract patients before surgeries (MD = -0.49, 95% CI: [-0.64, -0.33]; P<0.05).

### IOPg of cataract patients with phacoemulsification

**Fig 5:**
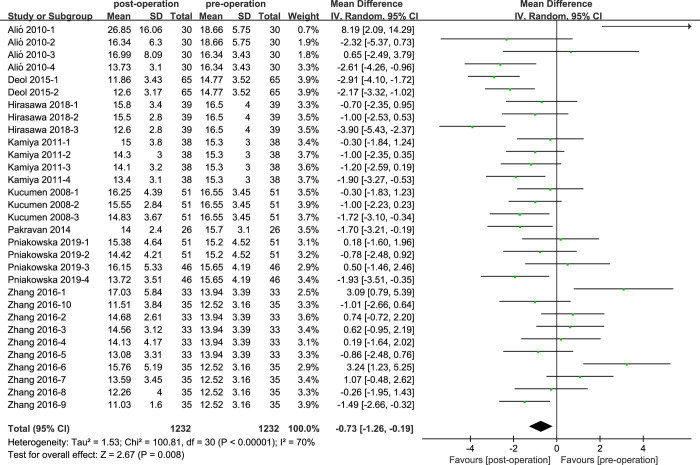
Comparison of IOPg values.

There were 8 studies reported the comparison of IOPg values in cataract subjects (Fig 5). Comparing the IOPg values, the result demonstrated that the heterogeneity (I^2^ = 70%; P<0.05) was high, and IOPg of postoperative cataract patients was lower than control group patients (MD = -0.73, 95% CI: [-1.26, -0.19]; P<0.05).

### IOPcc of cataract patients with phacoemulsification

**Fig 6:**
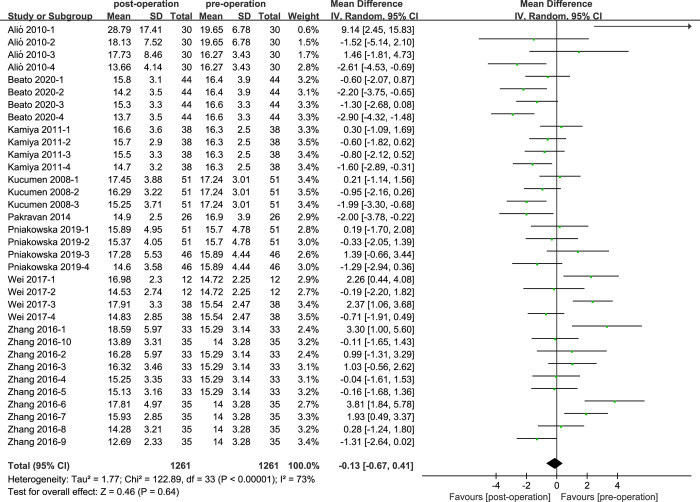
Comparison of IOPcc values.

Eight studies of IOPcc values of cataract patients were reported in the (Fig 6). The heterogeneity of IOPcc values was high (I^2^ = 73%; P<0.05). According to the statistical results, the IOPcc values between pre-operation and post-operation cataract patients did not show statistical difference (MD = -0.13, 95% CI: [-0.67, 0.41]; P = 0.64).

#### Subgroup analysis

We performed subgroup analysis from four aspects, including the type of clinical study, the size of corneal incision, the type of machine and the mode of operation (Table 2). For the CCT values, the subgroup analysis of the type of included studies delivered that ‘Retrospective clinical study’ could slightly reduce the heterogeneity(I^2^ = 51%), while the group of ‘Prospective clinical study’ did not decrease the heterogeneity. In terms of the corneal incision size, both the incision size of ‘2.4mm’ and ‘2.8mm’ groups showed low heterogeneity(I^2^_2.4_ = 0%; I^2^_2.8_ = 18%), the incision size of ‘2.2mm’ showed moderate(I^2^_2.2_ = 29%). The other corneal incision size all showed high heterogeneity(I^2^>50%). For the type of measurement machines, the heterogeneity of ‘Pentacam’ obviously decreased(I^2^ = 0%), while the heterogeneity of ‘Corvis ST’ was still high(I^2^ = 64%). The different mode of operation showed high heterogeneity(I^2^>50%).

**Table 2:** a. Subgroup analysis of CCT. b. Subgroup analysis of CH. c. Subgroup analysis of IOPg. d. Subgroup analysis of IOPcc.

					Heterogeneity			
		No.	MD (95%CI)	Q	I^2^	P_Q_	χ^2^	P
The type of clinical study	Prospective clinical study Retrospective clinical study	977 152	11.98(5.92, 18.03) 1.36 (-9.34, 12.05)	99.99 6.13	73% 51%	0.000 0.11	3.88 0.25	0.000 0.80
The size of corneal incision	2.2 2.4 2.75 2.8 3.0	165 162 258 269 275	23.00 (13.17, 32.83) 8.00(-0.60, 16.61) 14.84(-1.53, 31.21) 3.37(-2.67, 9.41) 8.40(-5.19, 22.00)	5.62 0.66 38.22 7.36 43.90	29% 0% 87% 18% 82%	0.23 0.96 0.000 0.29 0.000	4.59 1.82 1.78 1.09 1.21	0.000 0.07 0.08 0.27 0.23
The type of machine	ORA Corvis ST Pentacam	852 217 60	11.03 (3.43, 18.64) 10.13 (1.30, 18.97) 10.34 (-1.54, 22.21)	93.40 16.71 0.04	78% 64% 0%	0.000 0.01 0.98	2.84 2.25 1.71	0.004 0.02 0.09
The mode of operation	PHACO FLACS	1077 100	9.37(3.49, 15.25) 13.42(-1.99, 28.83)	97.97 13.92	71% 78%	0.000 0.003	3.12 1.71	0.002 0.09
					Heterogeneity			
		No.	MD(95%CI)	Q	I^2^	P_Q_	χ^2^	P
The type of clinical study	Prospective clinical study Retrospective clinical study	1091 152	-0.46(-0.67, -0.25) -0.36(-0.84, 0.11)	60.45 7.74	55% 61%	0.000 0.05	4.23 1.49	0.000 0.14
The size of corneal incision	1.8 2.0 2.2 2.4 2.75 2.8 3.0	60 194 165 153 318 158 175	-1.36(-2.62, -0.11) -0.83(-1.49, -0.18) -0.29(-0.68, 0.10) -0.17(-0.81, 0.47) -0.44(-0.89, 0.00) -0.14(-0.41, 0.14) -0.53(-0.92, -0.13)	1.30 9.47 5.58 7.24 24.19 4.11 2.91	23% 68% 28% 72% 71% 3% 0%	0.25 0.02 0.23 0.03 0.001 0.39 0.57	2.13 2.49 1.47 0.53 1.95 0.98 2.63	0.03 0.01 0.14 0.60 0.05 0.32 0.009
The type of machine	ORA Corvis ST Pentacam	1243 - -	-0.44 (-0.63,-0.25) - -	68.32 - -	55% - -	0.000 - -	4.54 - -	0.000 - -
					Heterogeneity			
		No.	MD (95%CI)	Q	I^2^	P_Q_	χ^**2**^	P
The type of clinical study	Prospective clinical study Retrospective clinical study	950 282	-0.46(-1.09, 0.17) -1.66 (-2.40, -0.92)	80.54 9.36	70% 47%	0.000 0.10	1.44 4.39	0.15 0.000
The size of corneal incision	1.8 2.0 2.2 2.4 2.75 2.8 3.0	60 194 165 153 60 295 175	2.59 (-7.69, 12.87) -0.60 (-1.69, 0.50) 0.60(-0.47, 1.68) -1.05(-1.84, -0.26) -1.27(-4.41, 1.88) -1.47(-2.21, -0.72) 0.21 (-1.34, 1.77)	9.11 4.71 7.81 1.83 3.23 14.16 19.35	89% 36% 49% 0% 69% 51% 79%	0.003 0.19 0.10 0.40 0.07 0.05 0.000	0.49 1.07 1.10 2.61 0.79 3.86 0.27	0.62 0.28 0.27 0.009 0.43 0.000 0.79
The type of machine	ORA Corvis ST	1115 117	-0.60 (-1.15, -0.05) -1.88 (-3.90, 0.14)	86.04 9.90	69% 80%	0.000 0.007	2.15 1.82	0.03 0.07
					Heterogeneity			
		No.	MD (95%CI)	Q	I^2^	P_Q_	χ^**2**^	P
The type of clinical study	Prospective clinical study Retrospective clinical study	1109 152	-0.02(-0.64, 0.59) -0.70 (-1.44, 0.04)	117.18 3.89	75% 23%	0.000 0.27	0.08 1.85	0.94 0.06
The size of corneal incision	1.8 2.0 2.2 2.4 2.75 2.8 3.0	60 194 165 153 236 178 275	3.42 (-6.99, 13.84) -0.12 (-1.19, 0.95) 0.83(-0.26, 1.91) -0.92(-2.13, 0.29) -1.65(-2.62, -0.67) -0.87(-1.61, -0.14) 0.87(-0.23, 1.97)	7.55 4.17 7.22 5.26 10.06 5.68 37.56	87% 28% 45% 62% 50% 30% 79%	0.006 0.24 0.12 0.07 0.07 0.22 0.000	0.64 0.23 1.50 1.49 3.30 2.34 1.55	0.52 0.82 0.13 0.14 0.001 0.02 0.12
The type of machine	ORA Corvis ST	1161 100	-0.29 (-0.60, 0.02) 0.93(-0.79, 2.65)	97.52 15.00	70% 80%	0.000 0.002	1.02 1.06	0.31 0.29
The mode of operation	PHACO FLACS	1209 100	-0.32(-0.86, 0.21) 0.93(-0.79, 2.65)	98.40 15.00	70% 80%	0.000 0.000	1.19 1.06	0.24 0.29

For the CH values, we performed three aspects on it. The heterogeneity of the group of ‘Prospective clinical study’ and ‘Retrospective clinical study’ did not show meaningful change (I^2^_prospective_ = 55%; I^2^_retrospective_ = 61%). For the size of corneal incision, the incision size of ‘1.8mm’, ‘2.2mm’, ‘2.8mm’ and ‘3.0mm’ showed low heterogeneity(I^2^_1.8_ = 23%; I^2^_2.2_ = 28%; I^2^_2.8_ = 3%; I^2^_3.0_ = 0%), while the size of ‘2.0mm’, ‘2.4mm’ and ‘2.75mm’ showed high heterogeneity(I^2^_2.0_ = 68%; I^2^_2.4_ = 72%; I^2^_2.75_ = 71%). The subgroup analysis of the type of machine did not show significant change(I^2^ = 55%), all included studies in this group only used ‘ORA’.

For the IOPg values, we conducted subgroup analysis according to the three aspects, except for the corneal incision size of ‘2.4mm’ showed low heterogeneity(I^2^ = 0%), the other results showed moderate or high heterogeneity(I^2^>25%). For the IOPcc values, we performed analysis through four aspects, while the results of subgroup analysis were similar with ‘IOPg’, only the heterogeneity of ‘Retrospective clinical study’ showed low heterogeneity, the heterogeneity of other groups was moderate or high.

#### Sensitivity analysis

**Fig 7:**
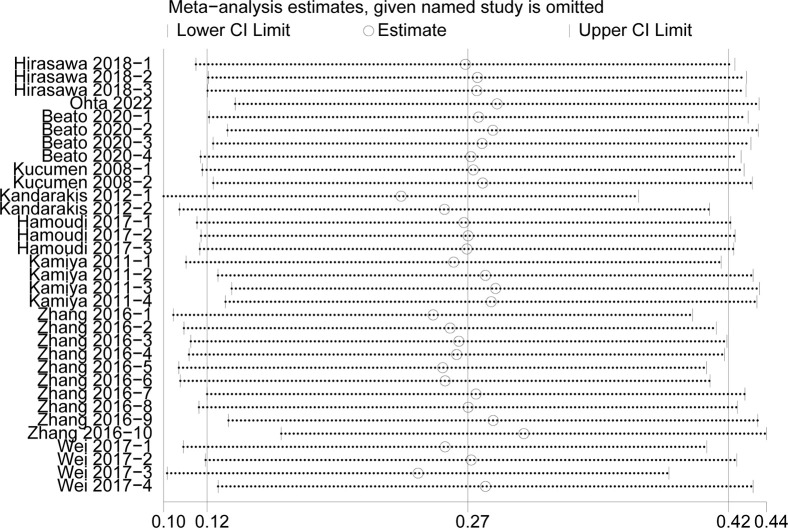
The sensitivity analysis of CCT.

**Fig 8:**
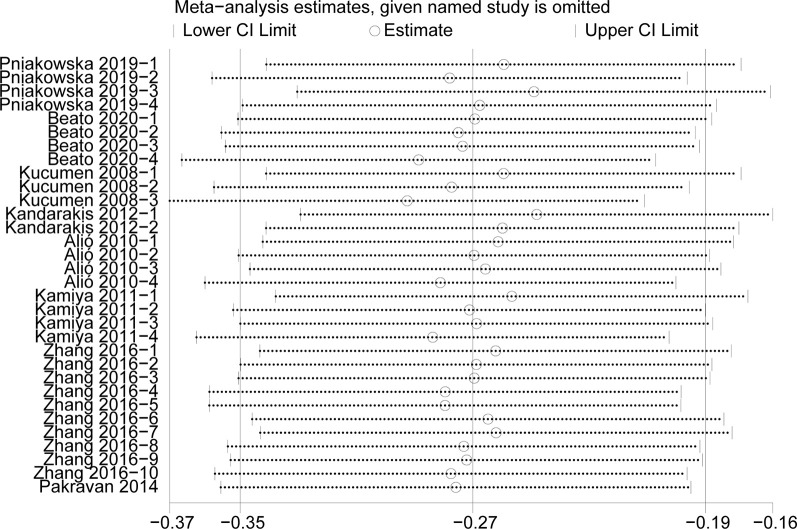
The sensitivity analysis of CH.

**Fig 9:**
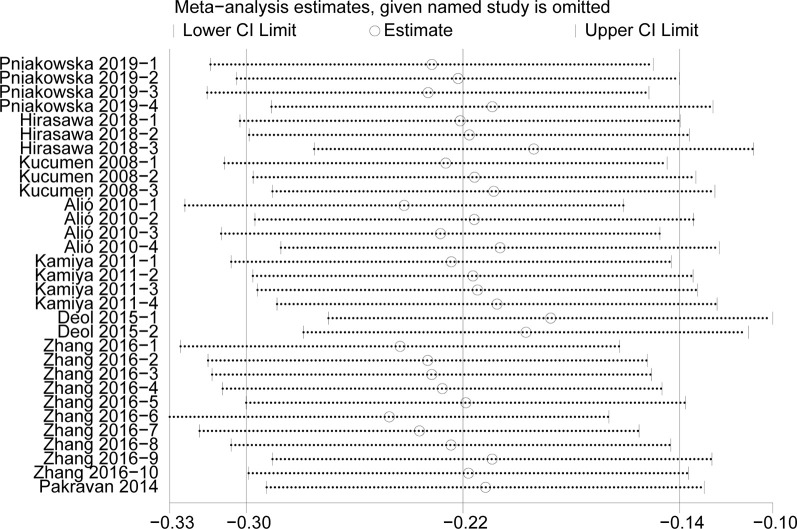
The sensitivity analysis of IOPg.

**Fig 10:**
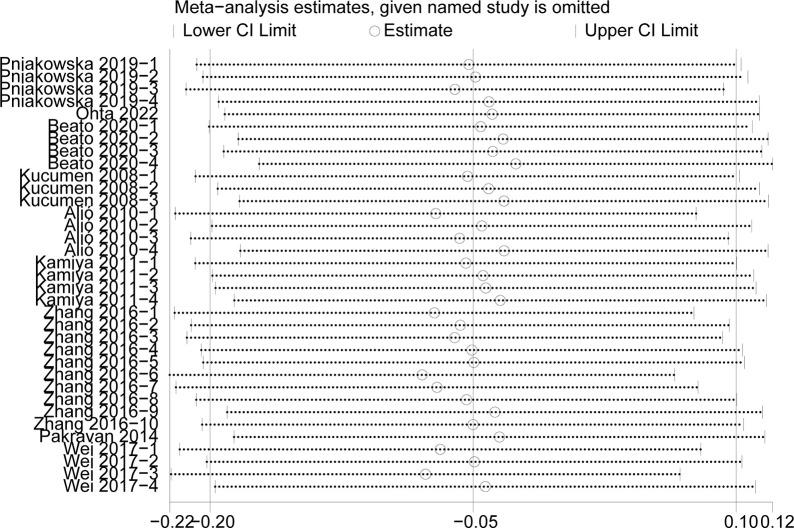
The sensitivity analysis of IOPcc.

We conducted the sensitivity analysis to assess the stability of the results of the CCT, CH, IOPg and IOPcc (heterogeneity: I^2^>50%) (Figs 7–10). The CRF values were excluded because of the low heterogeneity. The values of CCT, CH, IOPg and IOPcc were all in the effective range.

#### Publication bias

We conducted the tests of publication bias for the values of CCT, CH, IOPg and IOPcc(Table 3). According to the data analysis of Egger’s Test, the results showed statistically publication bias in control group and postoperative cataract patients of CH, IOPg and IOPcc(P< 0.05). The result of CCT did not show significant publication bias(P = 0.086).

**Table 3:** Publication bias.

	P
CCT	0.086
CH	0.020
IOPg	0.002
IOPcc	0.032

#### Quality assessment

All of the included articles were prospective or retrospective studies, we used the Newcastle-Ottawa Scale (NOS) items to assess the quality (Table 4). We evaluated the studies through 3 items: patient selection, comparability and outcome assessments. And the different scores showed various quality of researches, 7–9 indicated high quality, 4–6 indicated moderated quality, <4 indicated low quality [26]. Studies were ranked according to the star scoring scale, with higher scores indicating higher research quality. Seven studies showed high quality, and six studies showed moderated quality.

**Table 4:** Quality assessment.

Study	Patient selection	Comparability	Outcome assessments	Sum of score
Pniakowska, 2019 [13]	***	*	**	6
HIrasawa, 2018 [14]	***	*	***	7
Beato, 2020 [16]	***	*	***	7
Kucumen, 2008 [19]	***	*	***	7
Kandarakis, 2012 [20]	***	*	***	7
Hamoudi, 2017 [21]	***	*	***	7
Kamiya, 2011 [22]	***	*	***	7
Alió, 2010 [23]	***	*	**	6
Deol, 2015 [24]	***	*	***	7
Denoyer, 2013 [11]	***	*	**	6
Zhang, 2016 [17]	***	*	**	6
Pakravan, 2014 [18]	***	*	**	6
Wei, 2017 [25]	***	*	**	6

## Discussion

The current study aimed to assess the related corneal biomechanical change in cataract patients with phacoemulsification. This meta-analysis showed that during the initial period of postoperative, the values of CCT and IOPg statistically increased, the indexes gradually decreased. The final IOPg value was even lower than the preoperative IOPg during the observation period. According to the data displayed, the values of CH and CRF decreased, while the value of IOPcc did not show statistical difference between postoperative and control groups.

Various factors can cause the cataract, including aging, developmental abnormalities, metabolic disorders and trauma. Cataracts are the lens loses the optical clarity for any reasons. Phacoemulsification is a commonly surgical skill for cataract treatment. With the development of surgical skills, the corneal incision is becoming smaller. The healing process of corneal incision might influence the corneal biomechanical parameters. The change of postsurgical complication at initial postoperative time could make the parameters difference as well. Including the loss of corneal endothelial cells, corneal oedema, corneal decompensation and erosion, these factors might affect the corneal biomechanical properties.

In the meta-analysis, the results demonstrated that the values of CCT and IOPg in phacoemulsification groups were statistically higher than the control groups. Both CCT and IOPg values increased on the first surgical time, and gradually decreased on a period time. All subjects in the included studies were cataract patients with relative age range of the same levels. Surgical manipulations, such as instruments, surgical time and skills, phacoemulsification energy, can do direct or indirect harm to the corneal endothelium cells. And that might cause the corneal oedema and increase the CCT values. Although the IOPg values would rise after phacoemulsification surgery, it dropped back to a constant range within a certain period of time. In other words, the result indicated that the IOPg values stabilized beyond a certain cut-off period. Therefore, do not worry too much about constant deterioration, it might reverse instead. By all means, more complete and larger studies are needed.

The result displayed that the values of CH and CRF were statistically lower than the control groups. CH is a dynamic measurement of the viscous damping in the corneal tissue. And CH also represents the corneal energy absorption capability [17]. According to the data results, the CH values decreased on the first postoperative time, and then gradually increased. The reduction of CH values was associated with various factors. It has reported that corneal oedema could cause the CH reduction [19,27]. And the corneal incision and mechanical stress of phacoemulsification in the eye globe might be the possible elements as well [20]. The CRF is an indicator of the corneal resistance factor, it reflects the rigidity of corneal surface. Through the analysis of this study, the value of CRF decreased. The consequence showed no significant change of CRF value on the initial postoperative time. And the data changed after a period time. These changes might be associated with the healing of cornea and complication of surgery.

In the subgroup analysis, the type of included clinical studies did not show statistical difference of heterogeneity.

For the size of corneal incision, the heterogeneity of CCT and IOPg showed significantly decreased in ‘2.4mm’ group. The heterogeneity of CH decreased in ‘2.8mm’ and ‘3.0mm’ groups. There was no statistical difference in the size of corneal incision in the IOPcc group. For the machines used in the included studies, two studies used Corvis ST, one used Pentacam, and the others all used ORA, which made the result less convincing.

The result of quality assessment showed the high or moderate quality of included studies. NOS as an assessment tool which was evaluated by the researchers, there was inevitably subjective. The lack of methodological details in the published articles potentially limited inter-rater reliability, and caused the wrong assessment. At the same time, the different measured time of postoperative corneal biomechanics in various studies might affect the analysis results. The cross-comparison data of the same measurement time was too few that we could not explicitly compare the data in different articles.

The availability of a limited number of studies and the scarcity of data on the topic was one of the limitations of this article. More relevant data analysis could improve the confidence of the results. The mode of operation and limited measurement machines affect the result as well. Most studies used the conventional phacoemulsification surgical skills, only one study reported the FLACS. And only three articles used Corvis ST and Pentacam machines to evaluate the corneal biomechanical properties, most studies used ORA to measure the related indexes. Even though we try to performed subgroup analysis to decrease the high heterogeneity, the result did not show significant difference. The lack of sufficient data is relevant. According to the statistical analysis of publication bias, the result showed that there were significant publication bias in CH, IOPg and IOPcc. Even though we performed the sensitivity analysis to increase the reliability of the statistical result, however, the data of publication bias still might reduce the reliability of our result. These limitations underscore the necessary of more well-designed research to enhance the reliability of the relationship between phacoemulsification surgery and corneal biomechanics.

## Conclusion

We conducted a meta-analysis to evaluate the accessibility between phacoemulsification and corneal biomechanical characteristics. The result showed that the corneal biomechanics were associated with phacoemulsification. And the analysis results could provide some research ideas or evaluation indicators in the treatment process of cataract surgeries.

## Supporting information

S1 Checklist PRISMA 2020 checklist. (DOCX)
